# Development of a computer-tailored physical activity intervention for prostate and colorectal cancer patients and survivors: OncoActive

**DOI:** 10.1186/s12885-017-3397-z

**Published:** 2017-06-26

**Authors:** R. H. J. Golsteijn, C. Bolman, E. Volders, D. A. Peels, H. de Vries, L. Lechner

**Affiliations:** 1 Department of Psychology and Educational Sciences, Open University of the Netherlands, Heerlen, PO Box 2960, 6401 DL Heerlen, The Netherlands; 2 Department of Health Promotion, Maastricht University, Maastricht, The Netherlands

**Keywords:** Prostate cancer, Colorectal cancer, Physical activity, eHealth, Computer tailoring, Intervention mapping, Cancer survivorship

## Abstract

**Background:**

Cancer and cancer treatment coincide with substantial negative physical, psychological and psychosocial problems. Physical activity (PA) can positively affect the negative effects of cancer and cancer treatment and thereby increase quality of life in CPS. Nevertheless, only a minority of CPS meet PA guidelines. We developed the OncoActive (OncoActief in Dutch) intervention: a computer-tailored PA program to stimulate PA in prostate and colorectal CPS, because to our knowledge there are only a few PA interventions for these specific cancer types in the Netherlands

**Methods:**

The OncoActive intervention was developed through systematic adaptation of a proven effective, evidence-based, computer-tailored PA intervention for adults over fifty, called Active Plus. The Intervention Mapping (IM) protocol was used to guide the systematic adaptation. A literature study and interviews with prostate and colorectal CPS and health care professionals revealed that both general and cancer-specific PA determinants are important and should be addressed. Change objectives, theoretical methods and applications and the actual program content were adapted to address the specific needs, beliefs and cancer-related issues of prostate and colorectal CPS. Intervention participants received tailored PA advice three times, on internet and with printed materials, and a pedometer to set goals to improve PA. Pre- and pilot tests showed that the intervention was highly appreciated (target group) and regarded safe and feasible (healthcare professionals). The effectiveness of the intervention is being evaluated in a randomized controlled trial (RCT) (*n* = 428), consisting of an intervention group and a usual care waiting-list control group, with follow-up measurements at three, six and twelve months. Participants are recruited from seventeen hospitals and with posters, flyers and calls in several media.

**Discussion:**

Using the Intervention Mapping protocol resulted in a systematically adapted, theory and evidence-based intervention providing tailored PA advice to prostate and colorectal CPS. If the intervention turns out to be effective in increasing PA, as evaluated in a RCT, possibilities for nationwide implementation and extension to other cancer types will be explored.

**Trial registration:**

The study is registered in the Dutch Trial Register (NTR4296) on November 23rd 2013 and can be accessed at http://www.trialregister.nl/trialreg/admin/rctview.asp?TC=4296.

## Background

The number of newly diagnosed cancer patients and survivors (CPS) will increase significantly given the aging population and improved survival resulting from advances in early detection and cancer treatment [1, 2]. The growing population of CPS will pose increasing demands on healthcare, as cancer and cancer treatment coincide with substantial negative physical, psychological and psychosocial problems [3–11]. These problems can persist for years or even develop years after treatment. Interventions to reduce these negative effects of cancer and cancer treatment are therefore warranted.

Physical activity (PA) can positively affect the negative effects of cancer and cancer treatment and thereby increase quality of life in CPS [7, 12–21]. PA improves cardiorespiratory fitness and health-related quality of life (HRQoL), and reduces treatment-related side effects, fatigue, pain, distress, anxiety and depression both during and after active treatment [7, 13, 14, 19, 22, 23]. Some studies have even indicated that PA decreases cancer-specific and total mortality risk [24–26]. Besides these positive effects during and after active cancer treatment and on cancer recurrence and survival, being physically active is also important for CPS as they have a higher risk of developing second primary cancers and of developing comorbidities such as cardiovascular disease, diabetes and osteoporosis on which PA has a preventive effect [27].

Despite these benefits, and although PA is regarded as safe and feasible both during and after cancer treatment [12, 27, 28], only 30–47% of CPS meet PA guidelines [29, 30]. Moreover, PA behavior declines during treatment, and does not reach pre-treatment levels after completing treatment [21, 31]. Thus, interventions to stimulate PA are needed for this population

Diagnosis of cancer can be a ‘teachable moment’ for behavior change and a majority of CPS are interested in information about PA or participating in an exercise program [21, 32–37]. The majority prefers an unsupervised, home-based PA program, with walking as the preferred exercise mode [21, 34, 36, 38]. However, currently most PA programs in the Netherlands are hospital/healthcare-based, supervised exercise programs, aimed at sports. Although valuable, these programs are also demanding for both patients and health care professionals. An easily accessible, home-based PA program, aimed at stimulating PA in daily life and leisure time, offered at low costs and requiring minimal staff may offer a valuable alternative. Accordingly, we developed the OncoActive (OncoActief in Dutch) intervention: a computer-tailored PA program provided online and with printed materials. This paper describes the development process of the intervention, using the Intervention Mapping (IM) protocol and the design of a randomized controlled trial (RCT) to evaluate the effectiveness of the program. The intervention was targeted at prostate and colorectal CPS, because to our knowledge there are only a few PA interventions for these specific cancer types in the Netherlands [39–42]. More detailed rationale for the specific target population can be found in the methods section (needs assessment).

## Methods

The OncoActive intervention was developed through systematic adaptation of a proven effective, evidence-based, computer-tailored PA intervention for adults over fifty, called Active Plus [43, 44]. The Active Plus intervention has been delivered in either a print-based or a web-based version [45, 46]. Since the median age for a prostate or colorectal cancer diagnosis are 66 and 68 years respectively, and more than 96% of CPS are aged fifty and over [47], this intervention was assumed to be an ideal starting point. Computer-tailoring provides the opportunity to tailor the content to the specific needs of individual CPS. The IM protocol was used to adapt the intervention in a systematic way [48].

IM provides a systematic approach for the development of theory and evidence-based health promotion programs comprising six steps (Table 1). Although the IM protocol is primarily used to develop new interventions, the protocol is also useful for adapting evidence-based interventions for new target populations as is the case in our study. The protocol helps in finding a balance between containing the core elements of the original intervention while making it relevant for the new target population [48]. The application of these six steps for the development of the OncoActive intervention is briefly described below.

**Table 1:** Intervention mapping steps [48]

Step 1. Needs Assessment	*Assessing the health problem, its impact on quality of life and its related behavior*
Step 2. Program outcomes and objectives	*Adapting performance objectives, determinants and change objectives for the new target population*
Step 3. Program design	*Adapting theoretical methods and practical applications based on new change objectives or inadequate methods from the original intervention*
Step 4. Program production	*Adapting scope, sequence, materials and delivery channels and pretesting materials*
Step 5. Program implementation plan	*Developing an implementation plan for the new program*
Step 6. Evaluation	*Planning and implementing an effectiveness and process evaluation for the new program*

## Step 1: Needs assessment

The OncoActive intervention is aimed at prostate and colorectal CPS. Prostate and colorectal CPS represent a large proportion of the total CPS population in the Netherlands. Prostate cancer is the most common cancer site among Dutch men with 10,497 new cases in 2015, representing 19% of all newly diagnosed male cancer patients. Colorectal cancer is the second most common cancer site in both men and women in the Netherlands with 15,549 new cases in 2015, representing 15% of all newly diagnosed male and female cancer patients. Both cancer types have relatively high survival rates: a 5-year survival rate of 88–99% for prostate cancer and 62–65% for colorectal cancer [47, 49]. By selecting only two cancer types, we could better fine-tune the intervention to the specific needs and capabilities of prostate and colorectal CPS.

Cancer and cancer-treatment related side effects have a profound influence on quality of life. Although treatment improves survival rates, the inherent side effects have a negative influence on both physical and social functioning and thereby on quality of life [7, 17]. Prostate and colorectal CPS both experience some similar and some unique treatment related side effects. Decreased muscular strength, decreased physical fitness, functional limitations, bowel dysfunction, sexual dysfunction, altered body constitution, pain, fatigue, sleep disorders, emotional distress, depression, anxiety, fear of recurrence, challenges with body image and cognitive limitations are experienced in both cancer types. Urinary incontinence and hormonal treatment related side effects are more common in prostate cancer, while stoma related limitations, peripheral neuropathy and nausea are more common in colorectal cancer [3, 4, 6, 10, 11, 17, 50–57]. In particular, colorectal CPS have a higher risk of developing comorbidities such as type II diabetes and cardiovascular disease, second colorectal cancers and other primary cancers [7, 28, 58].

PA has consistently been shown to improve prostate and colorectal cancer treatment related side effects and thereby quality of life both during and after treatment [12–15, 17, 19, 20, 28, 51, 54, 56, 57, 59–61]. PA is also a preventive factor for the associated comorbidities and secondary/new cancers. As a result, PA guidelines for CPS have been established in several countries. International guidelines in general state that CPS should aim to be physically active (moderate to vigorous) for at least 150 min per week [62]. In the Netherlands CPS are advised to adhere (if possible) to the general Dutch PA guidelines, which require them to be physically active with moderate to vigorous intensity for at least 30 min a day on at least five days per week [63].

Only a minority of CPS adhere to PA guidelines. Adherence to PA guidelines for prostate CPS has been reported to vary between 29 and 47% [29, 30, 59, 64, 65] and is even lower in colorectal CPS: 20–40% [29–31, 51, 64, 66, 67]. PA levels are known to decline during treatment and do not reach pre-treatment levels after completing treatment [21, 68]. Thus, the majority do not take full advantage of the positive effects of PA during and after treatment, highlighting the need for an intervention to increase PA in the target group.

The negative effects of cancer and cancer treatment, the positive influence of PA on them and the low and decreasing adherence to PA guidelines already highlight the need for PA programs. Additionally, studies regarding supportive care needs have shown that CPS have a substantial perceived need for healthy lifestyle information and programs including PA [69–71]. According to the literature a majority of CPS are interested in information about PA or participating in a PA program [21, 33–35, 37, 38, 62]. As a result, the following program goals were formulated: Insufficiently active prostate and colorectal CPS become motivated to be physically active, initiate PA and maintain the newly attained PA level. Physically active prostate and colorectal CPS maintain or slightly increase their PA level.

In order to promote the desired behavior (i.e. being physically active) within the target population it is important to gain more insight into their specific motivating and hindering factors regarding the behavior and preferences in a PA program. Therefore, we systematically searched the literature regarding these topics. To confirm and expand this information we conducted interviews with our target group and healthcare professionals about PA advantages, cancer specific barriers to PA and information and intervention preferences regarding a computer-tailored intervention among our target group. We conducted twenty-nine semi-structured interviews with prostate (*n* = 18) and colorectal (*n* = 11) CPS and fifteen interviews with healthcare professionals (i.e. oncologist/urologist, physiologist, oncology nurse, oncology physiotherapist, oncology trainer) to explore the determinants of PA within the target group and their intervention preferences. Interviews were systematically analyzed with Qualicoder (www.qualicoder.com), according to the framework method [72]. By establishing such a planning group and thus involving the target group and healthcare professionals in the actual intervention development, we were able to take their wishes and preferences for the intervention into account. Findings from the interviews regarding the content of the intervention in relation to the findings from the literature are discussed in steps two and three (which concern determinants and intervention content).

## Step 2: Program outcomes and objectives

### Performance objectives

The main goal of the OncoActive intervention is to increase and maintain PA behavior of prostate and colorectal cancer CPS, as mentioned in Step1. Further specifying this health promoting behavior, in comparison with the original program, is the first task of Step 2 [48]. The original Active Plus intervention was aimed at increasing PA in two ways: by increasing and maintaining leisure time PA and by increasing and maintaining PA in people’s daily routines [73]. According to the literature influencing these PA behaviors is also relevant for, and preferred by prostate and colorectal CPS [21, 33, 38, 74, 75]. Subsequently specific health promoting behaviors are translated into performance objectives (POs). POs clarify what is expected from someone participating in the intervention and thus performing the desired health promoting behavior [48]. As the specific health promoting behaviors from the original Active Plus intervention are also relevant for prostate and colorectal CPS, the according POs can remain the same for the new target group. POs for the OncoActive intervention are mentioned in Table 2.

**Table 2:** Performance objectives for awareness raising, initiation and maintenance of PA among prostate and colorectal CPS

PO.1	*Prostate and colorectal CPS monitor their PA level*
PO.2	*Prostate and colorectal CPS indicate reasons to be physically active*
PO.3	*Prostate and colorectal CPS identify solutions to take away the barriers to be physically active*
PO.4	*Prostate and colorectal CPS decide to become more physically active*
PO.5	*Prostate and colorectal CPS make specific plans and set goals to become more physically active*
PO.6	*Prostate and colorectal CPS increase their PA*
PO.7	*Prostate and colorectal CPS make specific plans to cope with difficult situations occurring while being physically active*
PO.8	*Prostate and colorectal CPS maintain their PA level by enhancing their routine and preventing relapses*

*Note: PA includes recreational PA and PA in daily life*

### Determinants

Several studies regarding psychosocial determinants of PA in CPS have shown that attitude, subjective norms and perceived behavioral control (constructs of the Theory of Planned Behavior (TBP)) predict intention to engage in PA and PA behavior [68, 76–85]. Pinto and Ciccolo [77] reported that self-efficacy and outcome expectations (constructs of Social Cognitive Theory (SCT)) were important determinants of PA behavior. Higher self-efficacy is associated with more PA [21, 86, 87]. Furthermore, PA interventions based on the Transtheoretical Model (TTM), and thus tailored to the behavioral stage of change, proved to be a predictor of exercise adherence and to be effective in improving fitness, general health and reducing pain and fatigue in CPS [68, 88]. The I-Change model integrates these theories and models [89].

Based on the original Active Plus intervention [73], important psychological determinants are addressed in the OncoActive intervention ranging from pre-motivational determinants (e.g. awareness, knowledge and risk perception), motivational determinants (attitude, social influence beliefs, self-efficacy) and post-motivational determinants (goal setting, action planning) using input from the following social cognitive models: the I-Change Model [89–91] (a model integrating ideas of TPB [92], SCT [93], TTM [94], the Health Belief model [95] and goal setting theories [96, 97]), the Health Action Process Approach [98, 99], theories of self-regulation [100–102] and the Precaution Adoption Process Model [103]. An examination of the literature and interviews with the target group and health care providers regarding the benefits of PA and barriers to PA specifically for prostate and colorectal CPS were conducted to identify differences in the operationalization of the determinants.

#### Benefits of PA for prostate and colorectal CPS

In order to increase understanding and motivation of prostate and colorectal CPS towards PA, it is important to inform them about the benefits of PA as attitude is an important predictor of intention for PA [7, 34, 68, 77, 104]. Prove positive effects of PA during and after cancer treatment were identified by a systematic search of the literature and are listed in Table 3 Positive effects include improvements in both physical and mental aspects of health, as well as tertiary prevention of other chronic diseases [7, 19, 56, 60, 105–109].

**Table 3:** Benefits of and barriers to PA in prostate and colorectal CPS

*Benefits of PA*	
*Findings from literature* [7, 17, 19, 35, 56, 60, 105–109, 129–131, 135, 150–153]	*Findings from interviews* [110]
Increased: - physical functioning - muscle strength - quality of life - cardiorespiratory fitness - self-esteem - mood - incontinence - sense of achievement Decreased: - treatment related side effects - fatigue - anxiety - depression - distress - pain - insomnia Prevention of: - comorbidities - cancer recurrence - secondary cancers - cancer mortality	Perceived benefits CPS: - better physical fitness - better mental health - feeling better and healthier - being able to achieve goals - take mind off of cancer - better body weight Addition from healthcare professionals: - increased survival - reduced risk on comorbidities
*Barriers to PA*	
*Findings from literature* [7, 34, 35, 53, 82, 104, 129, 130–132, 135, 136, 150, 151, 153–156]	*Findings from interviews* [110]
General barriers: - bad weather - lack of time - lack of facilities - lack of support - motivational problems - financial costs - no enjoyment from PA - PA not a priority Cancer-specific barriers: - fatigue - decreased physical fitness - decreased muscle strength - pain - saving energy for treatments - infection risk - embarrassment about bodily changes - depression - fear of doing too much/injuries - symptoms from comorbidities - stoma - peripheral neuropathy - (urinary) incontinence or diarrhea - nausea and vomiting - cancer treatment	Prostate and colorectal CPS: - fatigue - pain - incontinence - peripheral neuropathy - lack of motivation - poor physical fitness - joint or muscle problems - lack of time - bad weather - stoma Healthcare professionals: - lymphedema - fear of movement - hand-foot syndrome (side effect from chemotherapy drugs for colorectal cancer) - problems with sitting on a bicycle saddle

The outcomes from the interviews with CPS and healthcare professionals (see Table 3) largely confirmed the findings from the literature. Although prostate and colorectal CPS did not mention benefits as specific as stated in the literature (for example, better mental health instead of less anxiety or depression), they perceived that PA had beneficial effects on their physical and mental health and enabled them to achieve goals in their daily life. Healthcare professionals additionally mentioned an increased survival and a reduction in the risk for comorbidities [110].

#### Barriers to PA for prostate and colorectal CPS

As illustrated in Table 3, according to the literature, both general and cancer-specific barriers can result in CPS not being physically active and should thus get special attention in a PA program [6, 51, 62, 104, 111, 112]. Physical complaints are often dependent on cancer type and the associated treatment. Physical complaints for colorectal CPS may include a stoma, peripheral neuropathy, (urinary) incontinence or diarrhea, nausea and vomiting [51], whereas urinary incontinence is the most important physical complaint in prostate CPS.

The findings from the literature were confirmed in the interviews, with fatigue, pain, incontinence and peripheral neuropathy being the most frequently mentioned barriers for being physically active. Besides cancer-specific barriers, the interviewed CPS also mentioned general barriers including lack of motivation, lack of time and bad weather [110]. Findings are listed in Table 3.

As barriers may prevent CPS from being physically active, it is important that a PA intervention for prostate and colorectal CPS pays special attention to the general barriers, but especially to the cancer-specific barriers. Providing suggestions to overcome the barriers could increase self-efficacy and perceived behavioral control, which are important predictors of intention for PA and actual PA behavior [68].

### Change objectives

Both performance objectives and the determinants that should be addressed are comparable to the original Active Plus intervention. Consequently, major changes in the general structure of the intervention were not regarded as necessary. Yet, findings from both interviews and the literature suggested that the content should also address cancer-specific topics. Determinants like attitude, knowledge and self-efficacy should be directed at the specific needs, beliefs and cancer related issues of CPS.

Therefore, we decided to add and/or adapt some change objectives to address these specific themes. For example, for the PO ‘prostate and colorectal CPS identify solutions to take away the barriers to being physically active’ combined with the determinant self-efficacy, we added the change objective ‘prostate and colorectal CPS feel confident about being able to take away and cope with cancer-specific barriers to being physically active’. Some other examples can be found in Table 4. Findings from the literature and interviews were also used in the production of the intervention content (see Step 4).

## Step 3: Program design

### Theoretical methods, practical applications and intervention preferences for CPS

Theoretical methods and practical applications are necessary to address the existing, adapted and added change objectives. In order to establish the adoption of an active lifestyle and maintenance of PA, it is important that behavior change techniques are incorporated in the intervention to improve PA behavior in CPS [7, 62]. We searched the literature and interviewed prostate and colorectal CPS regarding relevant theoretical methods and intervention content.

According to Pinto and Ciccolo [77], social-cognitive techniques for self-management, increasing self-efficacy, developing realistic outcome expectations, increasing intention and developing plans in line with motivational readiness are key concepts in a PA program for CPS. Modeling to increase self-efficacy, emphasizing benefits and fun (strengthening attitude) and informing significant others about the importance of PA (subjective norms) are important intervention components according to the Dutch cancer rehabilitation guideline [113].

According to the literature regarding the content that should be addressed with the theoretical methods and practical applications, CPS would like to receive information, advice and support regarding ways in which they can be physically active, both during and after treatment, the necessity to take special precautions due to illness and treatment, guidance in planning PA and giving notice to and emphasizing PA guidelines to increase awareness and acknowledge maintenance of PA [7, 34, 104]. Findings from our interviews indicated that it was important that a computer-tailored PA program (like the original Active Plus intervention, but adapted to CPS) provided guidance, ways to perform PA and emphasized PA benefits [110]. Healthcare providers suggested more practical things, like the use of graphic materials or videos, providing the possibility to consult with an expert or providing referral to an expert and using social media or apps.

### Theoretical methods and applications in the OncoActive intervention

To optimize participation of CPS in a PA program, it is important that an intervention is tailored to the patients’ interests, abilities, opportunities, and preferences [21, 35, 62]. Computer-tailoring provides the opportunity to easily adapt the intervention content to the specific characteristics of a patient to increase personal relevance. It is the core method of the OncoActive intervention (just as in the original Active Plus intervention). Computer tailoring is a method that uses questionnaires to assess characteristics, beliefs, behavior, etc., of the individual participants and automatically produces feedback. The feedback, based on the assessment, is created by using a message library and computer-based if-then algorithms to select the right messages. The feedback is personalized and automatically tailored to the personal characteristics of the participant and can thus also be tailored to cancer-specific needs and beliefs [114, 115]. Computer-tailoring was an effective method in changing PA behavior in the original Active Plus intervention [43, 44]. Several other studies and reviews also confirmed the effectiveness of computer tailoring in achieving behavioral change after providing tailored health promotion advice [114, 116–122].

Other theoretical methods used in the original Active Plus intervention included consciousness raising, self-monitoring, active learning, reinforcement, social modelling, persuasive communication and argumentation [45, 73]. These methods and the related practical applications can be retained for the OncoActive intervention. Additionally, theoretical methods and practical applications are also applied to the cancer specific content, as a result of the added and altered change objectives. Adding the change objective ‘Prostate and colorectal CPS learn about health benefits of PA related to cancer and can name personally relevant reasons for being sufficiently physically active’ requires that the practical strategies and content for attitude and knowledge should contain information about cancer-specific (perceived) benefits. A few other examples of the way we adapted the content to the prostate and colorectal CPS group can be found in Table 5. When applying a theoretical method it is important that the underlying theoretical conditions or parameters are respected [48]. For example, SCT [93] states that social modeling is only effective when the presentation of the methods meets certain conditions, such as participant identification with the model. For that reason, the existing role-model videos and pictures (for the paper-based version of the intervention) were replaced by videos and pictures with quotes of real cancer survivors instead of age and sex matched healthy adults.

Besides adjustments to methods and practical strategies regarding the cancer specific content, we also added some new applications based on the findings from the literature and our interviews. As self-efficacy is especially important [68, 123] in CPS, and the interviewed CPS and healthcare professionals mentioned the importance of the possibility to consult a professional, the option to consult a physical therapist with questions regarding PA and cancer was added to the intervention.

Although the original Active Plus intervention influenced PA behavior directly and path analyses showed that the intervention also influenced several determinants of PA, we looked for additional methods to enhance monitoring and goal setting to address the intention-behavior gap. Research in general [124–126] and specifically with CPS [127, 128] revealed that pedometers can be a valuable application for self-monitoring of PA behavior and goal setting. Therefore, we added the use of pedometers to the OncoActive intervention. By providing participants with instructions for monitoring, goal setting and adjusting goals, they are encouraged to self-regulate their PA behavior.

The described adaptations in methods and practical strategies were used to adapt existing and to develop new program components as described in the next section.

## Step 4: Program production

### Adaptation of program components

The adaptation and broadening of change objectives, theoretical methods and practical strategies also requires adaptation of program components. In general, all text messages were checked and if necessary adapted to relate them to the new target group of CPS. Additionally, intervention texts were edited and shortened by a professional editor. Some intervention elements were adapted more extensively and will be discussed below.

As mentioned in steps two and three, operationalization of the determinants for the OncoActive intervention was different from the original Active Plus intervention, as we added cancer-specific information regarding benefits of PA, attitude towards PA and difficult situations/barriers regarding PA. The change in determinants also requires adaptation in our screening instrument, in order to be able to tailor the new information to each individual CPS. As mentioned in step two, we searched the literature and used the information from the interviews to identify relevant pros, cons and barriers. This resulted in the addition of pros regarding PA being positively related to: better health, more energy/less fatigue, cancer recurrence, returning to ‘normal’ life, treatment related side effects, better bladder control and increased physical fitness [7, 35, 56, 109, 112, 129, 130]. Cons were added regarding PA being related to: increased fatigue, increased pain, increased lymphedema, higher risk of infection and hindering recovery from cancer [112, 130–134]. Difficult situations/barriers additionally included in the screening instrument and feedback library were urinary incontinence, feeling bad about bodily appearance, sleeping problems, being under treatment, suffering from treatment related side effects, lack of social support, peripheral neuropathy, afraid of falling, not knowing how much PA is allowed, fecal incontinence/diarrhea and having a stoma [7, 35, 129, 130, 133–136]. Some difficult situations, like feeling fatigued or feeling sad which are highly relevant for CPS were already included in the original Active Plus intervention.

Providing information on both the already included (general) and the cancer-specific pros/cons and difficult situation/barriers would result in an overload of information in the OncoActive intervention. Therefore, we decided to provide feedback on a maximum of seven pros, six cons and ten barriers. These were the same number of feedback messages that were given in the original intervention [45, 73]. As a result of this we had to apply a ranking to the delivered information. As cancer-specific determinants were expected to be of special relevance, we decided to provide feedback on these first. Complimentary feedback regarding the general determinants was provided until the maximum was reached or if there were no additional relevant determinants.

Another adaptation regarding the intervention materials involved the development of texts and information for using the pedometer for monitoring and goal setting. Tailored feedback messages regarding step goals were formulated and linked to the individual PA level of CPS. These messages also included instructions on how participants can continue on their own in setting new step goals once they have reached a goal. In addition to the tailored feedback, a brochure was provided with schemes CPS could use to keep track of their progress regarding their daily step count. The content was also translated into an interactive module on the website, to guide CPS in setting new step goals and monitoring their average daily step count.

As already mentioned in step three, role model videos and pictures of age and sex matched healthy older adults were replaced by pictures with quotes and video content from real cancer survivors. For this new content we conducted video-taped interviews with several cancer survivors. After filming the interviews, the content of the interviews was reviewed and short fragments with suitable quotes were added to the intervention. Colorectal CPS were shown videos/pictures of both (younger and older) males and females, whereas prostate CPS were only shown videos of (younger and older) males. These fragments showed for example which barriers the cancer survivors experienced and how they managed to overcome these barriers.

Based on the results of the interviews with CPS and health care providers, we also developed a module on the website in which CPS within the OncoActive intervention could consult a physical therapist with questions regarding PA, thus allowing them to receive a personal response to problems or difficulties. This module also contained a list with example questions and responses as a frequently asked questions database (FAQ). Participants were encouraged to look at these FAQ. Newly asked questions from participants were added (anonymized) to the ‘database’. The aim of this module was to enhance the self-efficacy of CPS to become physically active.

### Adaptation of delivery channels

The original Active Plus intervention was developed in a print-based version (exclusively in print materials, no additional website) [73] and a web-based version (exclusively online, no additional print materials) [45]. However, based on in-depth analyses it was suggested that for optimal effects the best solution would probably be to provide both delivery modes and giving the participant the choice of their preferred delivery mode [46, 137, 138]. Additionally, process evaluation data showed that in the original Active Plus intervention the print materials were used more often and better appreciated [139]. Taking into account these findings we decided to deliver the OncoActive intervention both printed and online alongside each other. In this way people could choose their own preferred delivery channel and web-based materials were supplemented with print-based material for every participant in order to optimize use and appreciation.

**Fig. 1:**
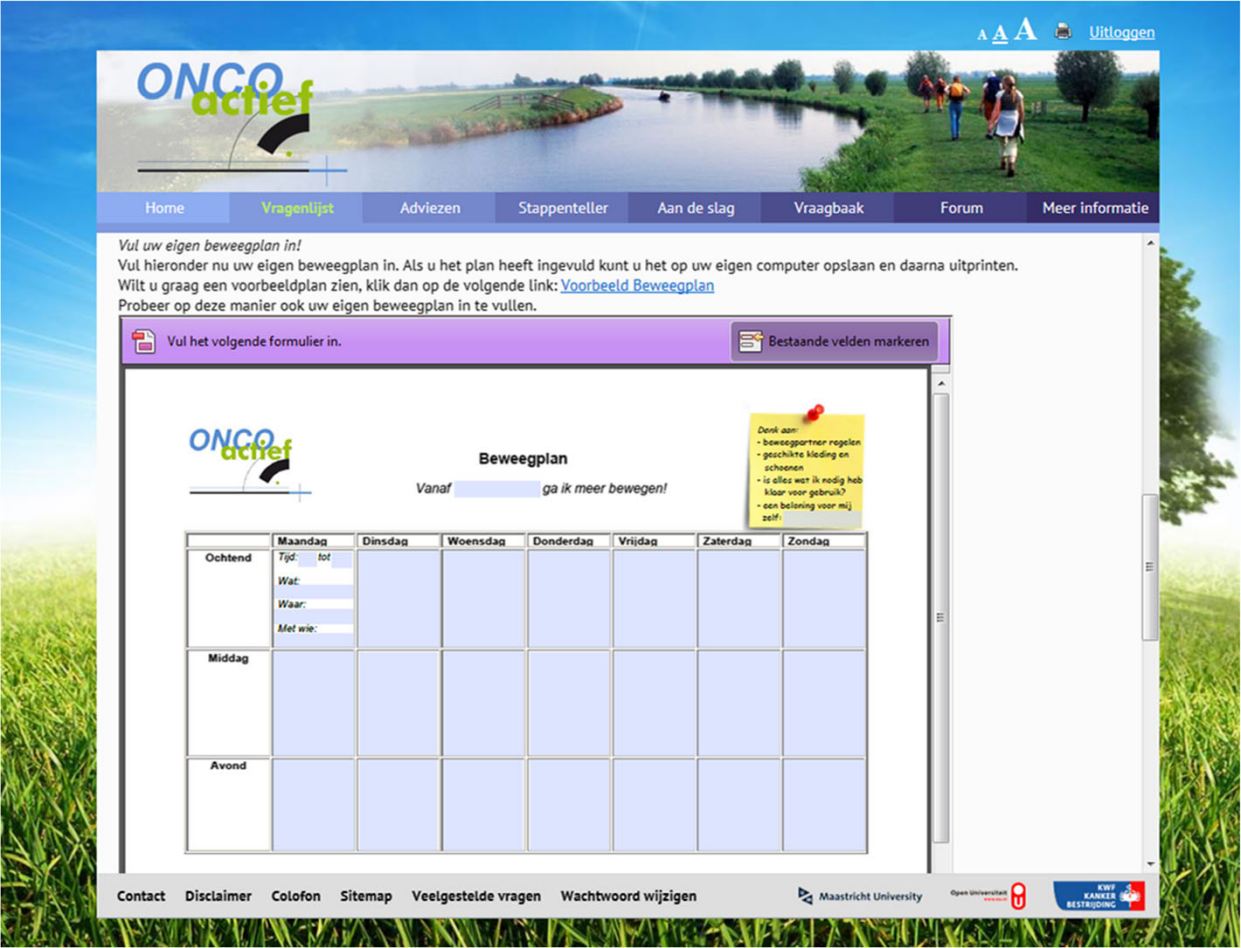
OncoActive website with integrated intervention materials

Process evaluation data of the original Active Plus intervention additionally indicated that access to the web-based intervention itself and to the web-based intervention materials should be simplified [139]. To simplify web access, we used URL’s automatically logging people into the right place on the website in e-mails inviting participants to visit the website. Intervention materials were more integrated in the website, as shown in Fig. 1. By integrating forms in this way, participants could start to fill out the form immediately, in contrast to the original Active Plus intervention. Additionally the website was constructed differently to increase the accessibility of the intervention content.

In order to keep participants more involved by visiting the website, we periodically provided them with additional news items, encouraging them to revisit the website. In total three news items were provided. The content and timing is described below.

### The intervention

The adaptation process described above resulted in the adapted OncoActive intervention. As explained in the previous sections the intervention is based on behavior change techniques and aimed at increasing awareness of PA behavior and stimulating PA during leisure time and in daily activities. Intervention participants receive tailored advice at three time points.

#### First advice

Participants receive their first advice within two weeks after completing the first questionnaire. The content is based on their answers to this questionnaire. Together with the advice they receive a pedometer (for own use) to monitor their PA behavior and to continually set goals to increase their PA.

#### Second advice

The second ‘follow-up’ advice, which participants receive two months after their first advice, is also based on answers to the first questionnaire. The content of both the first and the second advice is tailored to the behavioral stage of change according to the TTM: topics shown in Table 6, were addressed either in advice one or advice two depending on the stage of change at baseline. The content of the messages was tailored to cancer type and phase (i.e. during or after active treatment).

#### Third advice

Three months after the first questionnaire participants receive a new questionnaire and subsequently, within two weeks after completion, a third tailored advice. This final advice addresses changes in PA and PA related determinants since the start of the program. Improvements are rewarded, whereas suggestions for improvement are given in case of stagnation or decline.

#### News updates

Additionally, participants receive two or three news updates with extra information by e-mail. The first news update addresses the topic of incontinence and pelvic floor therapy and contains videos in which a pelvic floor therapist provided information. Participants suffering from urinary or fecal incontinence receive an e-mail that there is new content on the website, one month after their first advice.

The second news update contains video content in which a physical therapist explains the importance of PA during and after cancer treatment. All participants receive an e-mail to draw their attention to the new content on the website, six weeks after their first advice.

**Fig. 2:**
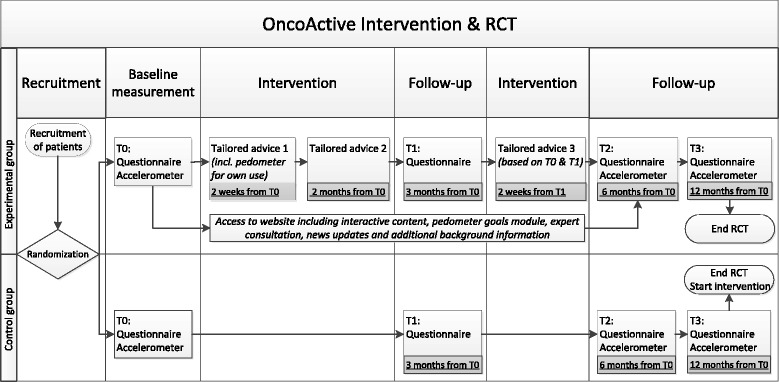
Schematic overview of the intervention and the associated randomized controlled trial

The third news update reminds participants about using their pedometer and provides them with tips and tricks to collect additional steps during their daily routines. All participants receive a notifying e-mail six weeks after their third (and last) tailored advice. A schematic overview of the intervention is shown in Fig. 2

#### Delivery channel

As previously mentioned, CPS can participate in the intervention both online and via paper-based questionnaires and advice. Every participant receives both log-in details for the OncoActive website to fill out the questionnaire and a paper-and-pencil version of the questionnaire. After completion of the questionnaire of their own choice, they receive their tailored advice both on the website and by normal mail. On the website they can also find additional interactive content (e.g. role model videos, home exercise instruction videos), a module for goal setting using the pedometer, the option to consult a physical therapist and additional information. A summary of intervention content and the addressed topics can be found in Table 6.

### Pretest and pilot-test

**Fig. 3:**
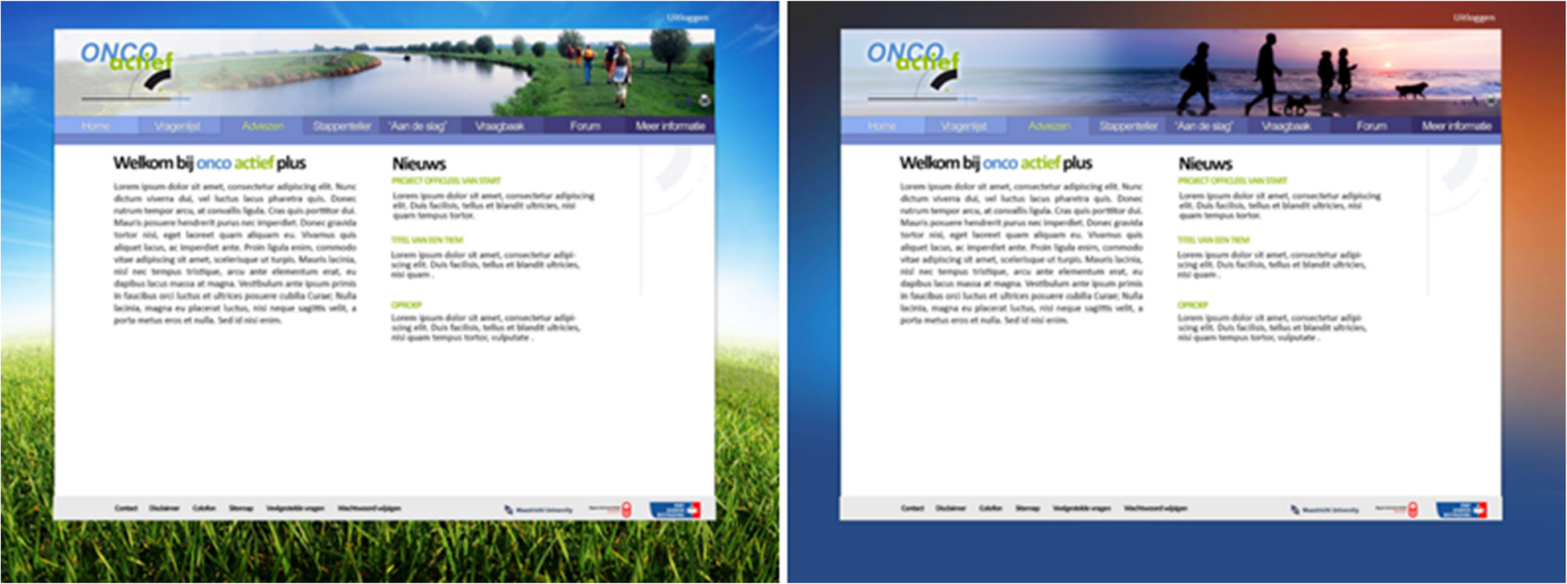
Potential website designs (design one on left) for the OncoActive intervention

As several intervention components were already evaluated within the Active Plus intervention, firstly we pretested newly developed intervention materials among twenty-nine CPS (who also participated in the interviews). We evaluated two possible designs for the websites (see Fig. 3). Design one was significantly more appealing and more appreciated (appeal: 3.7 vs. 3.2 on a 1–5 scale, *p* = .005; appreciation 7.5 vs. 6.6 on a 1–10 scale, *p* = .003). Furthermore, the pedometer, a role model video with a cancer survivor and the discussion group were appreciated as well (7.2, 7.7 and 7.0 respectively on a 1–10 scale) and valued as useful (3.7, 3.8 and 3.5 respectively on a 1–5 scale). Text messages for cancer specific barriers were rated 7.0 to 7.5 (on a 1–10) scale, except the text message about being physically active with a stoma, which scored a 5.6. To address this low score, we decided to add a brochure about PA with a stoma, developed by the Dutch stoma association, to the advice. Minor adaptations on the other text messages were made based on the suggestions of CPS.

After finishing intervention development, the complete intervention was evaluated in a small scale pilot study, in which the intervention was delivered to twenty-one CPS in a shortened time frame (i.e. two months instead of four months). CPS were recruited from one hospital and one radiotherapy institute. Findings from this pilot-test showed that the tailored advice was appreciated (7.5, 7.5 and 7.8 respectively on a 1–10 scale), as was the intervention overall (8.3 on a 1–10 scale) [140].The pedometer and cancer specific role model stories (i.e. new intervention components) were highly appreciated (8.5 and 7.7 on a 1–10 scale) and regarded as useful (4.2 and 3.9 on a 1–5 scale), especially the pedometer [140]. The newly developed website’s usability was evaluated using the System Usability Scale [141] and scored a 68.86 on this scale. According to this scale a score of 68 can be seen as average. Website components, i.e. the consultation of a physical therapist and additional background information were also appreciated (7.3 and 8.8 on a 1–10 scale) and regarded as useful (3.7 and 4.6 on a 1–5 scale). Lastly we also evaluated self-reported PA. Although we did not find a significant pre- to post-test increase in the minutes of moderate to vigorous PA, we found (even in the short time period) a significant increase in the number of days CPS reported being physically active for at least 30 min (3.8 vs. 5.3, *p* = .005).

As the intervention and the newly developed components received good scores on the pilot test, we decided not to adapt these components. In the pilot we tried to use a Facebook group as a discussion group. However, as this was not broadly used in the pilot study and because it was difficult to guarantee the privacy of the participants, as well as being difficult to integrate a Facebook group on the website, we decided to use a normal discussion forum for the final intervention. Additionally we noticed that participants had difficulties with filling out some parts of the questionnaires, such as the treatments they received and the social support and modeling they received from fellow CPS. Therefore, we decided to ask questions about received treatments together with a question about the type of cancer (i.e. prostate or colorectal) in a small questionnaire added to the informed consent form. In this way we had the opportunity to clarify ambiguities, in order to be assured that the participants received advice that matched their personal situation. With regard to the questions about social support and modeling from fellow CPS, we decided to drop this from the interventions, as it turned out that participants often did not know fellow CPS very well.

Finally, we also pretested the safety and feasibility of the content with cancer care professionals (*n* = 11) who also participated in the interviews. The scores in Table 7 show that the intervention content was regarded as highly feasible and safe. Minor adaptations (i.e. framing of a sentence) were made to the intervention texts based on suggestions of the cancer care professionals.

**Table 7:** Expert rating of the intervention content regarding safety and feasibility

Topics	Mean (SD) (scale 1–5)
Medical information is accurate	4.1 ± 0.8
PA recommendations are safe and suitable	4.4 ± 0.7
Sufficient safety precautions are taken	4.3 ± 0.9
Suitable for patients currently undergoing treatment	4.3 ± 0.6
Suitable for patients who finished treatment	4.4 ± 0.5
Information fits logic, language & experience of patients	4.7 ± 0.5

**Table 4:** Examples of change objectives added or altered for the OncoActive intervention

Performance objectives	Determinants				
	Awareness	Knowledge	Attitude	Self-efficacy	Action planning
1. PCa & CRC CPS monitor their physical activity level	Existing: PCa & CRC CPS become aware of their own PA level	*Old: OA know the PA recommendations and learn how to compare their own PA level with the recommendations*			
	Existing: PCa & CRC CPS monitor and report their own PA level	New: PCa & CRC CPS know the PA recommendations during and after cancer treatment and learn how to compare their own PA level with the recommendations			
2. PCa & CRC CPS indicate reasons to be physically active	Existing: PCA & CRC CPS become aware of their personally relevant benefits of being sufficiently physically active	Existing: PCa & CRC CPS learn about the general health benefits of sufficient PA and can name personal relevant reasons for being sufficiently physically active	Existing: PCa & CRC CPS feel positive about being sufficiently physically active		
		Added: PCa & CRC CPS learn about health benefits of PA related to cancer and can name personal relevant reasons for being sufficiently physically active			
3. PCa & CRC CPS identify solutions to take away the barriers to being physically active	Existing: PCa & CRC CPS become aware of situations and barriers that prevent them from being sufficiently physically active	Existing: PCa & CRC CPS learn how to identify general difficult situations and learn about solutions that can take away the barriers		Existing: PCa & CRC CPS feel confident about being able to take away and to cope with general barriers	
		Added: PCa & CRC CPS learn how to identify cancer-specific difficult situations and learn about solutions that can take away the barriers		Added: PCa & CRC CPS feel confident about being able to take away and cope with cancer-specific barriers	
				Added: PCA & CRC CPS feel confident about being able to cope with physical complaints due to cancer or cancer-treatment.	
5. PCa and CRC CPS make specific plans to become more physically active	Existing: PCa & CRC CPS become aware of the importance to make plans to increase their PA	Existing: PCa & CRC CPS learn how to make specific plans to increase their PA	Existing: PCa & CRC CPS feel positive about making plans to increase their PA	Existing: PCA & CRC CPS feel confident about making plans to increase their PA	Existing: PCA & CRC CPS make specific plans to increase their PA
				Existing: PCA & CRC CPS feel confident in being able to achieve their plans to increase their PA	Existing PCA & CRC CPS set goals to increase their PA

*PCa* prostate cancer, *CRC* colorectal cancer, *CPS* cancer patients and survivors, *OA* older adults, *PA* physical activity

**Table 5:** Examples of adaptations in theoretical methods, practical strategies and tools used in Active Plus and OncoActive

Personal determinant	Theoretical method	Practical strategy	Tools	
			Active plus	OncoActive
Awareness	Self-monitoring	Encourage monitoring of own behavior	Self-complete logbooks to monitor own PA behavior in last week.	Using a pedometer to monitor own PA behavior. (added)
Knowledge	Tailored feedback and information delivery	Provide tailored feedback about PA recommendations, PA benefits and PA possibilities	Computer-tailored feedback in text about PA recommendations, health benefits of sufficient PA and PA possibilities (recreational, daily PA)	Computer-tailored feedback about cancer-specific PA recommendations, health benefits and possibilities. (added)
Attitude	Feedback and argumentation	Provide personal feedback and arguments about pros and cons	Computer-tailored feedback in text on perceived positive and negative consequen-ces of PA. New argu-ments to change opi-nions are provided in text.	Computer-tailored feedback in text on perceived cancer-specific positive and negative consequences of PA. (added)
	Reinforcement	Provide ipsative feedback on changes in attitude: evaluation of changes	Computer-tailored feedback in text on positive changes in attitude towards PA at follow-up.	Computer-tailored feedback in text on cancer-specific changes in attitude towards PA at follow-up. (added)
Self-efficacy	Feedback and argumentation	Provide personal feedback and new arguments on self-efficacy	Computer-tailored feedback in text on difficult situations. New arguments to cope with these situations are provided.	Computer-tailored feedback in text on cancer-specific difficult situations and physical complaints. New arguments to cope with these situations. (added)
	Reinforcement	Provide ipsative feedback on changes in self-efficacy: evaluation of changes	Computer-tailored feedback in text on positive changes in perceptions of difficult situations at follow-up.	Computer-tailored feedback in text on cancer-specific positive changes in perceptions of difficult situations at follow-up. (added)
	Social modelling	Provide role model stories about difficult situations and how to cope	Picture/Video of similar others (same age and sex) with quotes about a similar perceived difficult situation and how the role model coped.	Picture/video of similar others (prostate or colorectal cancer survivor) with quotes about cancer-specific difficult situations and how the role model coped. (altered)
Action planning	Goal setting	Encourage to set PA behavior goals	Computer-tailored feedback in text about setting goals to be physically active for an extra number of minutes per week.	Computer-tailored feedback in text about setting goals to increase or maintain PA using a (provided) pedometer. (added)

**Table 6:** *Content summary of the OncoActive intervention*

Topics computer-tailored advice^a^	Summary of content^a^
*Advice 1 & 2*	
Awareness	- Graph with own behavior and guideline behavior
Knowledge	- Information regarding guideline - Information regarding positive effects of PA for prostate and colorectal CPS
Attitude	- Computer-tailored reflection and explanation on perceived pros and cons of PA
Motivation	- Role model video/picture about most important motivation for being physically active - Space to write down own (intrinsic) motivation for PA
Self-efficacy	- Computer-tailored reflection and explanation on perceived barriers and physical complaints - Suggestions to overcome barriers and deal with physical complaints - Role model video/picture demonstrating how to deal with barriers
PA suggestions	- Practical suggestions to be physically active according to the CPS’ preferences - Information about walking and cycling routes - Cancer-specific PA suggestions (e.g. PA groups for CPS) - Home exercises (video/pictures)
Goal setting	- Instructions about goal setting and monitoring using a pedometer
Action planning	- Scheme to plan PA on a weekly basis
Coping planning	- Scheme to construct if-then solutions for barriers or situations in which PA is difficult
Social support	- Encourage CPS to ask for support from their social environment - Suggestions to find someone to be physically active with
*Advice 3*	
Ipsative feedback	Feedback on: - Changes in PA behavior, activities and goals - Changes in health related factors (fatigue, quality of life) - Changes in PA determinants (intention, attitude, self-efficacy) - Changes in social support
Monitoring behavior	- Scheme to keep track of own PA behavior - Encouragement to continue pedometer use
Website components	Explanation
Pedometer module	Module for registering pedometer step counts to monitor PA behavior and set new step goals
Video content	Role model videos in which real cancer survivors talk about their own experiences and coping. Instruction videos with home exercises.
Expert consultation and FAQ	Module in which CPS can consult a physical therapist with questions regarding PA. Frequently asked questions are also shown.
Discussion group	Online discussion group in which CPS can exchange information, experiences and questions
Background information	Complementary information regarding nutrition, return to work, other website and interesting mobile applications
News update message	News messages regarding pelvic floor therapy, expert opinion about PA and cancer and tips and tricks to increase PA using a pedometer

^a^Sequence and content of topics are adjusted to the stage of change of the CPS

## Step 5: Program implementation plan

For implementation of the OncoActive intervention in a RCT, we created a network of hospitals and radiotherapy institutes in the Netherlands, including the two who participated in the small scale pilot. Contact persons within these institutions were surgeons, oncologists, urologists, research nurses and nurse practitioners. Seventeen hospitals agreed to participate in the active recruitment of CPS. Another five hospitals were not able to provide enough resources to actively recruit CPS, but agreed to distribute posters and flyers. Other reasons for not participating in the recruitment were the presence of (too many) other research projects and that the hospital treated only a few patients who met inclusion criteria.

Additionally daily and weekly regional newspapers, relevant websites and discussion groups were contacted to publish a call for CPS.

## Step 6: Evaluation plan

The final step entailed the development of a plan for the effect and process evaluation of the intervention. For this evaluation we compared an intervention group receiving the OncoActive intervention (who had also access to all usual care) to a usual care only control group in a RCT. The latter group had access to all usual care and received the OncoActive intervention after completion of all research measurements. Participants who provided informed consent to participate were randomly assigned to one of two study arms. The RCT was approved by the Medical Ethics Committee of the Zuyderland hospital (NL47678.096.14) and is registered in the Dutch Trial Register (NTR4296).

### Participants

CPS (≥18 years) diagnosed with colorectal or prostate cancer could participate in the trial if they were undergoing treatment with a curative intent, or if they successfully completed primary treatment (surgery, chemotherapy or radiation) up to one year ago. Surgery should have taken place at least 6 weeks before the start of the study. CPS with severe medical, psychiatric or cognitive illness which could interfere with participation in a PA program were excluded from participation. Proficient Dutch reading and speaking skills were required for the completion of questionnaires and reading the tailored advice.

### Power calculation

Sample size calculations were based on the outcomes of the previous studies on the effects of the Active Plus intervention. These studies found an effect size of 0.3 and effects were assumed to be comparable in CPS. Calculations showed that approximately 300 participants were needed for the effect study, based on this effect size, a power of .80 with an alpha of.05 and a correction for multilevel analyses (intracluster correlation coefficient = .005, design effect = 1.15). Drop-out was expected to be around 30% during the study, thus 428 participants were needed for enrollment at baseline.

### Design and procedure

Prostate and colorectal CPS were recruited from urology and/or oncology departments of seventeen hospitals in 2015 and 2016. Eligible CPS were identified by hospital staff and verbally informed (either in person or by telephone) about the research. Written information was handed over or sent by mail if the patient agreed to receive this information package. Additionally CPS were recruited with posters and flyers in non-participating hospitals, as well as with calls in local newspapers and on relevant websites and discussion groups. Participants responding to these messages were informed by the researchers and were also sent an information package by mail.

The information package included a letter with information about the study, a time schedule of the study, an informed-consent form and a pre-paid return envelope. Reminders were sent to participants if there was no response on the initial information package. CPS who agreed to participate, were randomized into one of the two research conditions as depicted in Fig. 2. Subsequently they were mailed an accelerometer with instructions to wear it for seven days. After wearing the accelerometer they received a questionnaire both online and on paper, with the choice to fill out one of them. After completing this baseline questionnaire (T0), the intervention group received the OncoActive intervention. Both groups had to fill out follow-up questionnaires at three time points: three (T1), six (T2) and twelve (T3) months after baseline. Participants were also requested to wear the accelerometer the week before they filled out T2 and T3 questionnaires. The control group received the OncoActive intervention after completing the last measurement (T3).

### Measurements

The primary outcome for this study was PA behavior, assessed both objectively with an accelerometer (Activity Monitor GT3X-BT Actigraph, Pensacola, Florida, US) and a validated self-report questionnaire (Short questionnaire to assess health-enhancing physical activity (SQUASH)) [142]. Secondary outcome measures included fatigue [143], anxiety and depression [144, 145], mental adjustment to cancer [146], quality of life [147] and health care consumption. Besides primary and secondary outcomes, CPS were also asked questions about demographics, cancer related characteristics (type of cancer, type of treatment currently undergoing/finished/planned for the near future), PA related determinants (awareness of personal PA level, attitude, self-efficacy, intention toward PA, habit strength). For the purpose of a process evaluation, participants of the intervention group were asked additional questions about use, appreciation, usefulness, readability, attractiveness, personal relevance and understanding of OncoActive. Besides the questionnaires, the use of the website and all accompanying elements were logged during the intervention period.

## Discussion

The purpose of this paper was to describe the systematic development process of the OncoActive intervention, a computer-tailored PA program for prostate and colorectal CPS both during and after treatment. The OncoActive intervention was aimed at increasing PA of prostate and colorectal CPS. By increasing PA behavior, the intervention may have a positive influence on cancer recovery and prevent other health problems. OncoActive was based on a proven-effective and evidence-based intervention for adults over fifty, the Active Plus intervention [43, 44]. Systematic adaptation of this intervention to the new target group was guided by the IM protocol [48].

In the first step we identified that only a minority of prostate and colorectal CPS adhered to PA guidelines, even though PA has the potential to positively influence health problems and address the decreased quality of life resulting from their disease and their treatment. In step two we identified the importance to address the cancer-specific determinants of PA as they differ from the determinants in a general population of adults over fifty. In step three we added theoretical methods and practical applications to address the cancer-specific determinants. Methods like a pedometer for goal setting and monitoring were added based on the findings from the literature and our interviews. In step four the actual program was developed and pre- and pilot tests revealed a high appreciation from the target group. The implementation and evaluation plan were described in steps five and six.

IM proved to be a useful approach for translating an existing intervention to a new target group. The use of this systematic approach in the intervention development increases the likelihood of OncoActive still being effective in increasing PA behavior and meeting the needs and preference of the new target group [48]. Major strengths of using IM include the possibility to retain the core elements of the original, proven effective [43, 44] intervention and the use of behavioral change theories and scientific literature. The involvement of prostate and colorectal CPS, at three time points (i.e. interviews, pretest and pilot test), and health care professionals was also regarded as a strength in the development of the OncoActive intervention. As a result, the intervention content is assumed to fit the needs and preferences of the target group. This was preliminarily confirmed by the findings of the small scale pilot study in which the intervention as a whole and its elements received positive evaluations from the target group. In particular, the newly added pedometer was identified as useful. Pre-posttest analyses even revealed an increase in PA behavior.

One of the major challenges in adapting an existing intervention to a new target group was to constrain the amount of information provided to the participants. By adding cancer-specific content to the already existing content, texts inevitably become longer. A lot of written information might particularly be a problem for lower educated participants [148]. To avoid an overload of information, we decided to give preference to cancer-specific information as mentioned in step four. Additionally, intervention texts were edited and shortened by a professional editor. Furthermore, participants were able to revisit the website as many times as they wanted during the intervention period and as they received a printed version of their advice, they could easily stop and return or re-read the information.

Strengths of the OncoActive intervention itself include the fact that CPS can participate from their own home and at their own preferred time, as was indicated as a preference of CPS in previous research [21, 34, 38]. Therefore, the intervention is regarded as easily accessible for the target group. Additionally, as both an online version and printed materials are provided, CPS can choose which delivery channel they prefer, which is suggested to increase the reach of the OncoActive intervention [46]. As the OncoActive intervention is based on the concept of computer-tailoring, the information regarding PA could be made more personally relevant. Information perceived as personally relevant is assumed to be read more often and processed more thoughtfully, increasing the likelihood of behavior change or maintenance [120, 149]. With time and place not being an issue, and the use of an automated process like computer tailoring, the OncoActive intervention has the potential to reach a large group of CPS with minimal resources in terms of personnel, and can thus be offered at low costs once it has been developed.

Notwithstanding the potential strengths, a RCT should still provide further insight into the effectiveness of the OncoActive intervention. This RCT will also provide insight into the question of whether a systematically adapted version of an effective intervention is still effective for a different target group. If the OncoActive intervention indeed proves to be effective in increasing PA, an implementation study for future nationwide implementation would be the next logical step. Information on optimal conditions (hindering and facilitating factors) for implementation will be derived from interviews with representatives of organizations relevant for implementation. Furthermore, if proven effective, the content of the OncoActive intervention can be extended to the cancer-specific determinants of other cancer types.

## Abbreviations

CPS: Cancer patients and survivors
CRC: Colorectal cancer
FAQ: Frequently asked questions
HRQoL: Health-related quality of life
IM: Intervention mapping
OA: Older adults
PA: Physical activity
PCa: Prostate cancer
PO: Performance objective
RCT: Randomized controlled trial
SCT: Social cognitive theory
TPB: Theory of planned behavior
TTM: Transtheoretical model
